# Criterion validity of ActiGraph monitoring devices for step counting and distance measurement in adults and older adults: a systematic review

**DOI:** 10.1186/s12984-022-01085-5

**Published:** 2022-10-17

**Authors:** Armelle-Myriane Ngueleu, Corentin Barthod, Krista Lynn Best, François Routhier, Martin Otis, Charles Sèbiyo Batcho

**Affiliations:** 1 Centre for Interdisciplinary Research in Rehabilitation and Social Integration, Centre intégré universitaire de santé et de services sociaux de la Capitale-Nationale, Québec City, Québec Canada; 2 Department of Rehabilitation, Faculty of Medicine, Université Laval, Québec City, Québec Canada; 3 Automation and Interactive Robotic Laboratory (AIRL), Department of Applied Science, Université de Quebec À Chicoutimi, 555 Blvd of University, Chicoutimi, Québec Canada

**Keywords:** Step counting, Distance, Adults, ActiGraph, Older adults

## Abstract

**Background:**

Wearable activity monitors such as ActiGraph monitoring devices are widely used, especially in research settings. Various research studies have assessed the criterion validity of ActiGraph devices for step counting and distance estimation in adults and older adults. Although several studies have used the ActiGraph devices as a reference system for activity monitoring, there is no summarized evidence of the psychometric properties. The main objective of this systematic review was to summarize evidence related to the criterion validity of ActiGraph monitoring devices for step counting and distance estimation in adults and/or older adults.

**Methods:**

Literature searches were conducted in six databases (Medline (OVID), Embase, IEEExplore, CINAHL, Engineering Village and Web of Science). Two reviewers independently conducted selection, a quality analysis of articles (using COSMIN and MacDermid’s grids) and data extraction.

**Results:**

This review included 21 studies involving 637 participants (age 30.3 ± 7.5 years (for adults) and 82.7 ± 3.3 years (for older adults)). Five ActiGraph devices (7164, GT1M, wGTX +, GT3X +/wGT3X + and wGT3X − BT) were used to collect data at the hip, wrist and ankle to assess various walking and running speeds (ranging from 0.2 m/s to 4.44 m/s) over durations of 2 min to 3 days (13 h 30 mins per day) for step counting and distance estimation. The ActiGraph GT3X +/wGT3X + and wGT3X − BT had better criterion validity than the ActiGraph 7164, wGTX + and GT1M according to walking and running speeds for step counting. Validity of ActiGraph wGT3X + was good for distance estimation.

**Conclusion:**

The ActiGraph wGT3X − BT and GT3X +/wGT3X + have good criterion validity for step counting, under certain conditions related to walking speeds, positioning and data processing.

**Supplementary Information:**

The online version contains supplementary material available at 10.1186/s12984-022-01085-5.

## Background

Mobility is essential to everyday life, with significant positive impacts on active aging, physical activity and quality of life in older adults [1]. Conversely, impaired mobility is an early predictor of physical disability [2]. Mobility can be achieved through various motor actions, such as locomotion (e.g. walking, running) in ambulatory people or displacement using manual wheelchair in people with physical disabilities [3, 4]. Walking is reported as the first form of locomotion in which people engage worldwide [5]. For most individuals, walking forms the foundation for maintaining mobility and also contributes substantially to daily physical activity (through active transportation, activities of daily living and exercise), which is important for health [6, 7]. It is low-cost and accessible to most people and can be easily incorporated into everyday life. Running is also a low-cost form of physical activity for those who are able to achieve it.

In clinical settings, many common rehabilitation measures (e.g., timed walking tests, balance tests) are used to assess components of ambulatory capacity. However, many tests of ambulatory capacity have floor effects that limit their responsiveness to detect changes in frail older adults [8]. The World Health Organization recommends using performance measures to determine impact of disease in daily life, and to avoid floor and ceiling effects that are often related to capacity tests [9]. Thus, wearable devices, which provide objective measures, are commonly used for walking performance assessment in research and clinical settings.

Locomotor activities can be measured by estimating distance travelled or by quantifying number of steps (e.g., walking or running) [10]. With the growing interest in the development of technological innovations, many easily wearable devices offer the possibility to obtain these locomotion-based parameters (e.g., distance, number of steps) during walking or running in daily life [11]. Among these devices, ActiGraph is one of the most commonly used activity monitors for research in various populations [12, 13]. ActiGraph is used to quantify the volume of walking (e.g., step count and distance) in people with incomplete spinal cord injury [14], hospitalized elderly [15, 16], stroke survivors [17–19], children aged 10–17 years [20, 21], people with multiple sclerosis [22, 23], or in healthy people [24–27]. There are several models of ActiGraph that, vary according to type of sensors (e.g., accelerometer, gyroscope) and data processing (e.g., filter). Knowing that walking speed may vary between elderly people and adults, it would be interesting to determine if walking speed affects the results accuracy. In the literature, studies have reported walking speeds affected outcomes accuracy for step counting and distance estimation [28–30]. Furthermore, results can be affected by positioning and ActiGraph devices [31–33].

Although several studies have used the ActiGraph as a reference system for step counting [21, 34–38], little is known about the psychometric properties (e.g., criterion validity). Criterion validity is an estimate of the extent to which a measure agrees with a gold standard. A recent systematic review has shown reliability and criterion validity of commercially available wearable devices for step counting, energy expenditure and heart rate but some devices such as ActiGraph were excluded due to the unmanageable number of returned studies following title and abstract screening [39]. Full et al. [39] reported that 133 studies out of 169 were performed in healthy people. To the best of our knowledge, no systematic review has been conducted regarding the criterion validity of ActiGraph in adults (less than 65 years) or older adults (65 years and more). The aim of this systematic review was: (1) to summarize evidence related to the criterion validity of ActiGraph devices for step counting and distance travelled in adults or older adults (2) to compare criterion validity of different devices of ActiGraph according to positioning, walking speed and various processing data in adults or older adults.

## Methods

This systematic review followed the “Preferred Reporting Items for Systematic Reviews and Meta-Analyses (PRISMA)” guidelines [40].

### Description of the ActiGraph devices

ActiGraph devices (manufactured by ActiGraph LLC Pensacola, FL) are small and lightweight activity monitors (mass: 19–27 g; width: 33–39 mm; height: 11–37 mm; thickness: 18–46 mm) that are equipped with an accelerometer and sometimes also with a gyroscope. The accelerometer measures linear acceleration in one or three orthogonal directions. ActiGraph detects dynamic accelerations (resulting from motion) ranging from ± 3 to ± 16 g and static acceleration (e.g., force of gravity detected when stationary) depending on device types [41]. The acceleration signal is digitized by an 8- and 12-bit analog-to-digital converter at a frequency of 10–100 Hz (in multiple of 10 Hz, e.g., 10 Hz, 20 Hz) depending on the device, filtered and reported as an "activity count". The signal is filtered at bandwidth of 0.21–2.5 Hz using a band-pass filter. Actilife software is a post-processed environment which determines steps per epoch. There are different ActiGraph devices: the ActiGraph 7164, the ActiGraph GT1M, the ActiGraph wGTX +, the ActiGraph GT3X +/wGT3X +, the ActiGraph GT3X-BT, and the ActiGraph GT9X +. These devices differ according to filter, mechanism used by the sensors (e.g. Piezoelectric, microelectro-mechanical-system capacitive), battery life or addition of other sensors (e.g., gyroscope and magnetometer for the ActiGraph GT9X +). The ActiGraph wGT3X + differs from ActiGraph GT3X + by adding a specific function for heart rate monitoring. The price varies between US$325 (+ US$349 for the Actilife software) and US$1016 depending on the device. According to the manufacturer's recommendations, ActiGraph devices can be positioned using wristbands or elastic bands on the wrist, ankle, thigh and/or hip. Characteristics of ActiGraph types are presented in Table 1.

**Table 1:** Characteristics of ActiGraph devices

ActiGraph devices	Sensor types	Dynamic range	Sensitivity	Sampling frequency	Filter types	Analysis algorithms	Actilife version	Communication mode	Firmware version
7161	Piezoelectric sensor (accelerometer)	0.05–2.13 g (1 g = 9.81 m/s^2^)	NR	10 Hz	Band-pass filter (0.21–2.28 Hz)	NR	NR	NR	NR
GT1M	Capacitive MEMS sensor (accelerometer)	± 5 g (0.05–2 g)	NR	30 Hz	Band-pass filter (bandwidth of 0.25–2.5 Hz)	Step count: per-epoch basis;	NR	NR	7.5.0
GT3X + /wGT3X +	Capacitive MEMS sensor (accelerometer)	± 3 g or ± 6 g	3 ng/LSB	30 to 100 Hz with step of 10	Band-pass filter (bandwidth of 0.25–2.5 Hz)	Step count: per-epoch basis;	ActiLife 6.13.3	Wireless	3.2.1 and 2.5.0 for GT3X + ; 2.5.0 for w GT3X + ;
wGT3X − BT	Capacitive MEMS sensor (accelerometer)	± 8 g	4 ng/LSB	30 to 100 Hz with step of 10	Band-pass filter (bandwidth of 0.25–2.5 Hz)	Step count: per-epoch basis;	ActiLife 6.12.0	Bluetooth	1.9.2
GT9X +	(accelerometer, gyroscope and magnetometer)	± 8 g and ± 16 g; ± 2000 deg/s; ± 4800 micro-Tesla	NR	30 to 100 Hz with step of 10	Band-pass filter (bandwidth of 0.25–2.5 Hz)	NR	ActiLife 6.11.5	Bluetooth	1.7.2

*MEMS* microelectromechanical system, *NR* non-reported

### Search strategy

For literature search, keywords were designed around three concepts, namely (#1) the activity monitor (“ActiGraph”), (#2) the psychometric property (“validity”) and, (#3) outcomes (“distance” or “step count”). A more detailed search strategy was then developed by AMN: Armelle-Myriane Ngueleu, and CB: Corentin Barthod, including key words related to the three basic concepts and their synonyms. The search strategy was conducted in each database: Medline (OVID), Embase, IEEExplore, CINAHL, Engineering Village and Web of Science according to free and controlled terminologies. The initial search was performed on February 15, 2020 and an update was performed on August 3, 2021.

### Study selection

To be included in this systematic review, the studies should have: (1) reported results pertaining to the criterion validity of an ActiGragh device, (2) analyzed variables for walking distance or step count, (3) used at least one of the following reference systems: motion capture, manual counting, video recording (for counting steps or distance), other valid device (for distance) or a predefined distance (for distance estimation), (4) included healthy participants (aged 18 and over) and (5) been published in English or French. Titles and abstract of the identified articles were screened independently by two reviewers (AMN, CB) who determined their eligibility. In case of discrepancies, consensus was reached through discussion. In the absence of consensus, a third reviewer (CSB: Charles Sèbiyo Batcho) screened the study and new discussions took place until consensus was reached. The same procedure was used for full-text selection.

### Methodological quality

The two reviewers (AMN, CB) independently conducted a quality analysis of the articles based on two quality assessment tools. First, the COSMIN grid (“consensus-based standards for the selection of health measurement instruments”) [42] was used to critically appraise the quality of the criterion validity [box H]). Second, MacDermid's grid was used to evaluate the structural and methodological qualities (research questions, study design, measurements, analyses and recommendations) of the articles [43]. These two grids provide information on the overall article quality. An initial meeting was held beforehand to agree between the two reviewers on each evaluation criterion. A second meeting was held following the independent critical appraisal by the two reviewers to reach consensus on the evaluation.

For each grid, the score was converted into a percentage. We assigned the value 1 to the point "excellent" and 0 to the points “good”, “fair” or “poor” for the COSMIN grid. The quality score for both grids was characterized as follows: Very low quality (VLQ) = 0–25%, low quality (LQ) = 26–50%, moderate quality (MQ) = 51–75% and high quality (HQ) = 76–100% [44]. Pre-consensus inter-rater agreement was calculated using the Gwet-weighted coefficient on each individual item of the COSMIN grid. The level of inter-rater agreement was defined as: poor < 0.0; slight 0.0–0.2; fair 0.21–0.4; moderate 0.41–0.6; substantial 0.61–0.8; excellent 0.81–1 [45]. An intraclass correlation coefficient (ICC) was calculated to assess inter-rater agreement on the overall McDermid grid score. The ICC score was defined as follows: values < 0.5 indicate poor agreement, values between 0.5 and 0.75 indicate moderate agreement, values between 0.76 and 0.9 indicate good agreement, and values between 0.91 and 1 indicate excellent agreement [46].

### Data extractions

Each reviewer performed a complete data extraction from the articles included in this review. The following targeted variables were extracted using a standard data extraction tool [43]: sample size, participants’ characteristics (age, body mass index), ActiGraph devices, ActiGraph positioning, reference systems, parameter evaluated (step count or distance), evaluation duration and validity index. The indices extracted for criterion validity were: accuracy (percentage), r (simple correlation coefficient), ICC (intraclass correlation coefficient), LoA (limit of agreement) and t-test.

### Data analysis

For studies reporting means comparison, a Cohen’s d (D) was calculated (see Eq. 1), in order to quantify the difference size as following:

1 $$D=\frac{Average of difference}{Standard deviation of gold standard}.$$

The criterion validity of the ActiGraph devices was determined using three interpretation tables depending on different indices (Pearson correlation coefficient, intra-class correlation coefficient and average comparisons). A measure was considered to be valid if it had a correlation qualified as “Good” or “Excellent” according to the magnitude r or ICC, or if the effect size was “trivial” according to Cohen’s d.

**Table:** 

The Pearson correlation coefficient is interpreted using the Cohen scale [47]	The intra-class correlation coefficient is interpreted using the Ciccetti scale [48]	The effect size (Cohen’s d) associated with average comparisons is interpreted using the Hopkins scale [49]
• < 0.3: Very low • Between 0.3 and 0.49: Moderate • Between 0.5 and 0.69: Good • Between 0.7 and 1: Excellent	• < 0.4: Very low • Between 0.4 and 0.59: low • Between 0.6 and 0.74: Good • Between 0.75 and 1: Excellent	< 0.2: Trivial Between 0.2 and 0.59: Low Between 0.6 and 1.19: Moderate Between 1.2 and 1.99: Important Between 2 and 4: Very important > 4: Extremely important

## Results

**Fig. 1:**
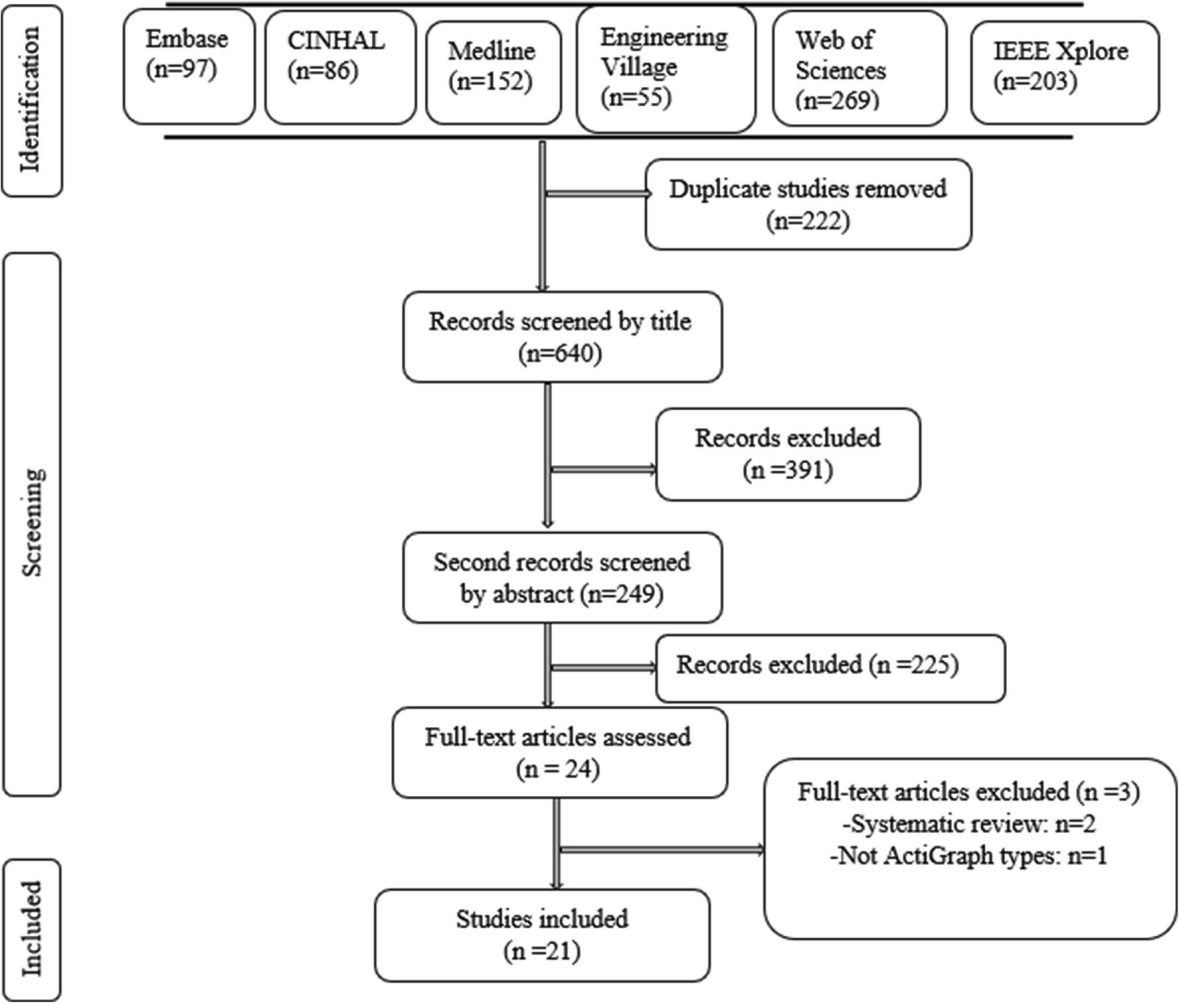
PRISMA flow chart for systematic review of the criterion validity of ActiGraph for step count and distance

Following the literature search, 862 articles were retrieved from the six databases and 21 articles were included after removal of duplicates, screening of titles, abstracts and full-text analysis of the articles (see Fig. 1) (Additional file 1).

### General characteristics of the studies

The total sample included 637 participants with an average age of 30.3 ± 7.5 years (for adults) and 82.7 ± 3.3 years (for older adults) (see Table 2). All the included articles reported the criterion validity of the ActiGraph devices for step counting. Only one article reported the criterion validity of ActiGraph type for both step counting and distance. Experiments were performed in older adults (n = 4) [1, 10, 16, 50] and in adults (n = 16) [11, 22, 24, 25, 27, 51–61]. In one study, assessment was performed in adults and older adults [26]. Reported walking speeds ranged from 0.43 to 4.43 m.s^−1^ [1, 10, 16, 22, 24–27, 50–54, 56, 57, 59–61]. Thirteen articles tested walking and running speeds in sessions lasting from 2 to 15 min [22, 24–27, 51–57, 59], of 30 min in a study [60] and one study used an incremental test (i.e., a speed that increased progressively) for two minutes [11]. In other studies, walking speeds were tested on walking distance of 10 [16], 40 [61] or 100 [1] meters, or for 100 steps [10]. Two studies have assessed an ActiGraph device during a full day (11.6 ± 1.5 h) [16] and three days (13 h 30 mins per day) [58], respectively. The handheld tally counter and video observation were used as gold standard in most studies except three papers that used StepWatch monitor as gold standard [16, 58, 61]. Experiments were performed in indoor setting in all studies except in two studies (outdoor setting) [58, 60].

**Table 2:** Characteristics of the included studies

Authors and year	Number of participants	Participants age: (mean ± standard deviation), year	Body mass index (mean ± standard deviation), kg/m^2^	ActiGraph types	Positioning of ActiGraph	ActiGraph orientation (attachment bracket)	Signal processing	Reference system used	Duration of assessment
Esliger et al. 2007 [52]	38	34.3 ± 18	26.2 ± 4.3	7164	Hip	Vertical in nylon pouch (belt)	NR	Manually counted	4 min
Abel Mark et al. 2008 [24]	20	29.4	NR	GT1M	Hip	Vertical (NR)	SF: 30 Hz	Manually counted	10 min
Sorti et al. 2008 [10]	34	79.2 ± 6.0	26.9 ± 4.1	NR	Hip	Lateral side of the hip (belt clip)	NR	Manually counted	100 steps
Motl et al. 2011 [22]	24	40.9	25.1	7164	Hip	Vertical (elastic belt)	SF:0.25–2.5 Hz; DR: 0.05–3.2G	Manually counted	6 min
Webber et al. 2014 [1]	35	81.5 ± 5.0	25.8	GT3X +	Hip	Anterior axillary line (elastic belt)	SF: 100 Hz;	Manually counted	100 m
Feito et al. 2015 [25]	22	23.8	NR	GT3X + and GT1M	Hip	Anterior axillary line (elastic belt)	NR	Manually counted	2 min
Lee et al. 2015 [27]	43	20.9 ± 1.9	25.5 ± 2.7	GT3X +	Hip	NR	SF: 30 Hz	Manually counted	3 min
Hickey et al. 2016 [54]	15	24.9	23.8	GT3X + and 7164	Hip	Anterior axillary line (elastic belt)	SF:0.25–2.5 Hz	Video recordings	5 min
Riel et al. 2016 [57]	30	27.9	23.6	GT3X +	Hip	Lateral on right anterior superior iliac spine (elastic belt)	SF: 100 Hz; DR: 8G	Manually counted	2 min
Webber et al. 2016 [16]	38	83.2 ± 7.1	NR	GT3X +	Hip and ankle	Anterior axillary line (strap)	NR	StepWatch monitor	10 m and full day (11.6 ± 1.5 h)
Chow et al. 2017 [51]	31	24	23.6	GT3X +	Hip and wrist	NR	SF: 30 Hz	Video recordings	3 min
Feng et al. 2017 [53]	25	25.9	NA	wGT3X − BT	Hip	NR (belt)	NR	Video recordings	4 min
Hochsmann et al. 2018 [26]	20	37.5	23.5	wGTX +	Hip and wrist	NR	NR	Video recordings	5 min
Jones et al. 2018 [56]	30	33	24.1	GT3X +	Hip	NR (elastic belt)	SF: 30 Hz	Manually counted	4 min
Imboden et al. 2018 [55]	30	49.2 ± 19.2	26.2 + -19.6	GT3X +	Hip	Anterior axillary line (elastic waistband)	SF: 60 Hz	Manually counted	2 to 15 min
Hergenroeder et al. 2018 [50]	43	87 ± 5.7	26.1 ± 4.1	GT3X +	Hip	Anterior aspect of the thigh (elastic belt)	NR	Manually counted	100 steps
Kendall et al. 2019 [11]	50	25.8	25,7	wGT3X − BT	Hip	NR (belt)	NR	Manually counted	2 min
Höchsmann et al. 2020 [58]	30	25	22	GT3X +	Hip and Wrist	NR	SF: 60 Hz	StepWatch monitor	3 days (13hrs30)
Bezuidenhout et al. 2021 [61]	30	42 ± 13	NR	GT3X +	Hip and ankle	Iliac crest-hip and proximal to the lateral malleolus-ankle	SF: 30 Hz	StepWatch monitor	40 m
Taoum et al. 2021 [60]	20	23 ± 3	22.7 ± 3.0	wGT3X +	Hip and Wrist	NR	SF: 30 Hz	Manually counted (step); GPS (distance)	Between 10 and 15 min; Total: 30 min
Karaca. et al. 2021 [59]	29	26.3 ± 6.2	24.07 ± 2.3	wGT3X − BT	Hip, Wrist and ankle	Mid-axillary line-hip (elastic belt); lateral side-wrist (band); lateral side-ankle (strap)	NR	Video recordings	2 min

*NR* non-reported, *SF* sampling frequency, *DR* dynamic range

### Devices of ActiGraph used

A total of five ActiGraph devices were reported in the studies including the ActiGraph 7164, the ActiGraph GT1M, the ActiGraph wGTX +, ActiGraph wGT3X − BT and the ActiGraph GT3X +. Among them, the ActiGraph 7164 (n = 3) and GT1M (n = 2) devices are unidirectional and the ActiGraph wGTX + (n = 1), wGT3X − BT (n = 3) and GT3X +/wGT3X + devices are tri-directional. The ActiGraph GT3X +/wGT3X + device was the most commonly used (n = 13). The positioning of the ActiGraph devices differed across studies: hip [1, 10, 11, 16, 22, 24, 25, 27, 50–57, 59–61], ankle [16, 59, 61] and wrist [51, 59, 60] (see Table 2). Fourteen studies positioned the ActiGraph devices only on the hip [1, 10, 11, 22, 24, 25, 27, 50, 52–57], four studies simultaneously on the hip and wrist [26, 51, 58, 60], two study on the hip and ankle [16, 61] and one study on the hip, wrist and ankle [59].

### Methodological quality

The scores on the MacDermid critical appraisal tool ranged from 50 to 91% with a mean ± SD of 74 ± 9.8% (see Table 3). Eleven articles were classified as high-quality, nine articles as moderate-quality, and one article as a low-quality. The results of the COSMIN grid are presented in Table 4 and the scores for criterion validity (box H) ranged from 50 to 100% with a mean of 74.8 ± 18.2%. Eleven articles were classified as high-quality, two articles as moderate-quality and eight articles as low-quality. All articles did not score for sample size except one study [11] and detailed exclusion/inclusion criteria, thus partially explaining the moderate quality score in both grids. The pre-consensus inter-rater agreement between reviewers for the total scores of the MacDermid and COSMIN grids was considered good (ICC = 0.87) and excellent (Gwet = 0.85–0.92), respectively.

**Table 3:** Assessment of methodological quality of studies using the MacDermid grid

Authors	MacDermid criteria (C)												Total score [22]	MacDermid percentage (%)	Quality (MacDermid)	Total inter-rater agreement
	C1	C2	C3	C4	C5	C6	C7	C8	C9	C10	C11	C12				
Esliger et al. 2007 [52]	2	1	1	2	0	NA	2	2	2	2	1	2	17	77%	HQ	94
Abel Mark et al. 2008 [24]	2	2	1	1	0	NA	2	2	1	2	2	1	16	73%	MQ	100
Sorti et al. 2008 [10]	2	2	2	2	0	NA	2	1	2	1	0	2	16	73%	MQ	100
Motl et al. 2011 [22]	1	2	2	1	0	NA	2	1	2	1	2	1	15	68%	MQ	94
Webber et al. 2014 [1]	2	2	1	2	0	NA	2	2	2	2	1	2	18	82%	HQ	100
Feito et al. 2015 [25]	2	2	2	2	0	NA	2	1	2	2	2	1	18	82%	HQ	79
Lee et al. 2015 [27]	2	1	1	2	0	NA	2	2	2	2	2	2	18	82%	HQ	100
Hickey et al. 2016 [54]	2	2	1	1	0	NA	2	1	2	1	2	2	16	73%	MQ	94
Riel et al. 2016 [57]	1	2	2	2	0	NA	2	2	2	2	2	1	18	82%	HQ	100
Webber et al. 2016 [16]	2	2	2	2	0	NA	2	2	2	2	2	2	20	91%	HQ	100
Chow et al. 2017 [51]	2	2	1	1	0	NA	2	1	1	2	1	1	14	64%	MQ	80
Feng et al. 2017 [53]	1	2	1	1	0	NA	1	1	1	1	1	1	11	50%	LQ	100
Hochsmann et al. 2018 [26]	2	1	2	1	0	NA	2	2	1	1	1	2	15	68%	MQ	100
Jones et al. 2018 [56]	2	2	2	1	0	NA	2	1	2	1	1	2	16	73%	MQ	81
Imboden et al. 2018 [55]	2	2	1	2	0	NA	1	1	2	1	0	2	14	64%	MQ	81
Hergenroeder et al. 2018 [50]	2	2	1	1	0	NA	2	2	1	2	1	1	15	68%	MQ	100
Kendall et al. 2019 [11]	2	2	1	2	1	NA	2	1	2	2	2	2	19	86%	HQ	75
Höchsmann et al. 2020 [58]	2	1	1	2	0	NA	2	2	2	2	2	2	18	82%	HQ	94
Bezuidenhout et al. 2021 [61]	1	2	1	2	0	NA	1	2	2	2	2	2	17	77%	HQ	84
Taoum et al. 2021 [60]	2	2	1	2	0	NA	2	2	2	1	2	2	18	82%	HQ	84
Karaca et al. 2021 [59]	2	2	1	2	0	NA	2	2	2	2	2	2	19	86%	HQ	94

*MQ* moderate quality, *LQ* low quality, *HQ* high quality, *NA* not applicable

**Table 4:** Assessment of studies examining criterion validity using COSMIN grid

Authors	Criteria (C)							TOTAL	COSMIN Percentage (%)	Quality (COSMIN)	Total inter-rater agreement
	C1	C2	C3	C4	C5	C6	C7				
Esliger et al. 2007 [52]	0	0	0	1	1	1	NA	3	50	LQ	83
Abel Mark et al. 2008 [24]	1	1	1	1	1	1	NA	6	100	HQ	100
Sorti et al. 2008 [10]	1	1	0	1	1	1	NA	5	83	HQ	83
Motl et al. 2011 [22]	1	0	1	1	1	1	NA	5	83	HQ	83
Webber et al. 2014 [1]	0	0	0	1	1	1	NA	3	50	LQ	83
Feito et al. 2015 [25]	1	1	0	1	1	1	NA	5	83	HQ	66
Lee et al. 2015 [27]	1	0	0	1	0	1	NA	3	50	LQ	83
Hickey et al. 2016 [54]	1	0	0	1	1	1	NA	4	67	MQ	83
Riel et al. 2016 [57]	1	1	1	1	1	1	NA	6	100	HQ	83
Webber et al. 2016 [16]	1	1	0	1	1	1	NA	5	83	HQ	100
Chow et al. 2017 [51]	0	1	1	1	1	1	NA	5	83	HQ	100
Feng et al. 2017 [53]	1	0	1	1	0	1	NA	4	67	MQ	83
Hochsmann et al. 2018 [26]	1	0	1	1	1	1	NA	5	83	HQ	83
Jones et al. 2018 [56]	1	0	1	1	1	1	NA	5	83	HQ	83
Imboden et al. 2018 [55]	1	0	0	1	0	1	NA	5	83	HQ	66
Hergenroeder et al. 2018 [50]	0	0	0	1	1	1	NA	3	50	LQ	100
Kendall et al. 2019 [11]	1	1	1	1	1	1	NA	6	100	HQ	83
Höchsmann et al. 2020 [58]	0	0	0	1	1	1	NA	3	50	LQ	66
Bezuidenhout et al. 2021 [61]	0	0	0	0	1	1	NA	2	33.33	LQ	83
Taoum et al. 2021 [60]	0	1	0	1	1	1	NA	4	67	LQ	100
Karaca et al. 2021 [59]	0	0	0	1	1	1	NA	3	50	LQ	100

*NA* not applicable, 1 for excellent, 0 for good or fair or poor, *MQ* moderate quality, *LQ* low quality, *HQ* high quality

### Criterion validity of ActiGraph devices for step counting

Twelve studies used manual step counting as gold standard [1, 10, 11, 22, 24, 25, 27, 50, 55–57, 60], six studies used video observation [26, 51–54, 59] and three studies used StepWatch monitor [16, 58, 61] (see Table 2). In terms of validity indices, five studies compared the ActiGraph and reference system measures by determining the Pearson/Spearman correlation coefficient [1, 24, 52, 56, 59] and six studies used an intra-class correlation coefficient [11, 16, 27, 57, 58, 61]. Fourteen studies used different tests of average comparison (confidence interval, standard error of measurement, percentage of difference, percent error, mean measurement bias scores, mixed linear model, mean absolute percentages error (MAPE), Bland–Altman plots, t-tests). The results are associated with walking speeds (see Table 5). Results of Cohen’s d for studies with average comparison are presented in Table 6.

**Table 5:** Criterion validity indices of ActiGraph types for step counting and distance in healthy adults and older adults

Authors	ActiGraph devices	Gold standard	Criterion validity indices	Outcomes (walking or running speeds)
Esliger et al. 2007 [52]	7164	Manually counted	Bland-Atlman plots, paried-sampled t-tests, Pearson correlation coefficients	5.3% difference (50 m/min) 0.008% difference (83 m/min) 0.006% difference (133 m/min)
Abel Mark et al. 2008 [24]	GT1M	Manually counted	Pearson correlation coefficient	− 0.37 (54 m/min) − 0.58 (80 m/min) − 0.69 (107 m/min) − 0.64 (134 m/min) − 0.58 (161 m/min) − 0.54 (188 m/min)
Sorti et al. 2008 [10]	NR	Manually counted	Mean absolute percent error (MAPE)	19.1% (< 0.8 m/s) 7.5% (≥ 0.8 m/s) 3.3% (≥ 1.0 m/s)
Motl et al. 2011 [22]	7164	Manually counted	Percentage of real number, percent error	97.2%; error: 2.8% (54 m/min) 100%; error: 0% (80 m/min) 96.6%; error: − 3.4% (107 m/min)
Webber et al. 2014 [1]	GT3X +	Manually counted	Percent error, unpaired t tests, Bland–Altman plots, Mean measurement bias scores, Spearman rank order correlation coefficients	23.2% (1.1 m/s)
Feito et al. 2015 [25]	GT3X + et GT1M	Manually counted	Percentage of difference	GT1M: − 61% (N); − 7% (LFE) GT3X: − 58% (N); − 4% (LFE) (40 m/min) GT1M: − 31% (N); − 1% (LFE) GT3X: − 31% (N); 1% (LFE) (54 m/min) GT1M: − 7% (N); − 2% (LFE) GT3X: − 6% (N); 2% (LFE) (67 m/min) GT1M: − 6% (N); − 2% (LFE) GT3X: − 1% (N); 3% (LFE) (80 m/min) GT1M: − 9% (N); − 2% (LFE) GT3X: − 2% (N); 3% (LFE) (94 m/min)
Lee et al. 2015 [27]	GT3X +	Manually counted	Intraclass correlation coefficients (ICC) with measures of consistency; Bland–Altman plots; standard error of measurement	0.29 CI: − 0.30 to 0.62 (54 m/min) 0.33 CI: − 0.25 to 0.63 (67 m/min) 0.61 CI: 0.28–0.79 (80 m/min) 0.99 CI: 0.98–0.99 (94 m/min) 0.99 CI: 0.98–0.99 (107 m/min)
Hickey et al. 2016 [54]	GT3X + et 7164	Video recordings	Percentage of difference, confidence interval (95%) (CI)	7164: − 13%; CI: − 19% to − 6% GT3X (N): − 54% CI: − 65% to − 42% GT3X (LFE): 1% CI: − 9% to 11% (2.4 km/h) 7164: − 5%; CI: − 6% to − 5% GT3X (N): − 2% CI: − 3% to − 2% GT3X (LFE): − 1% CI: − 1% to − 0.4% (4.8 km/h) 7164: − 5%; CI: − 6% to − 4% GT3X (N): − 2% CI − 2.6% to − 1.7% GT3X (LFE): − 1% CI: − 1% to − 0.4% (7.2 km/h) 7164: − 5% CI: − 6% to − 2% GT3X (N): − 3% CI: − 3% to − 2% GT3X (LFE): − 1% CI: − 2% to − 0.4% (9.7 km/h)
Riel et al. 2016 [57]	GT3X +	Manually counted	Intra-class correlation coefficient	0.03 CI: − 0.09 to − 0.21 (3.2 km/h) 0.55 CI: 0.13–0.78 (4.8 km/h) 0.64 CI: 0.16–0.84 (6.4 km/h)
Webber et al. 2016 [16]	GT3X +	StepWatch monitor	Intraclass correlation coefficients, Bland–Altman plots, Independent t tests	Ankle (LFE) 0.94 CI: 0.87–0.97 (0.4 ± 0.2 m/s) Ankle (N) 0.68 CI: − 0.21 to 0.89 (0.4 ± 0.2 m/s) Hip (LFE) 0.83 CI: 0.33–0.94 (0.4 ± 0.2 m/s) Hip (N) − 0.05 CI: − 0.19 to 0.15 (0.4 ± 0.2 m/s)
Chow et al. 2017 [51]	GT3X +	Video recordings	Percentage error	Hip: − 0.1% Wrist: − 28.9% (5 km/h) Hip: 0.9% Wrist: − 36.0% (6.5 km/h) Hip: − 2.4% Wrist: − 48.4% (8 km/h) Hip: − 0.1% Wrist: − 49.9% (10 km/h) Hip: 0.2% Wrist: − 50.0% (12 km/h)
Feng et al. 2017 [53]	wGT3X − BT	Video recordings	Percent error, confidence interval (95%)	− 4% CI: − 9% to 3% (0.9 m/s) − 2.5% CI: − 12% to 0.8% (1.1 m/s) − 0.3% CI: − 0.8% to 0.8% (1.3 m/s)
Hochsmann et al. 2018 [26]	wGTX +	Video recordings	Mean absolute percentages error (MAPE)	Hip: 82% Wrist: 47% (1.6 km/h) Hip: 24% Wrist: 22% (3.2 km/h) Hip: < 3% Wrist: 30% (4.8 km/h) Hip: < 3% Wrist: 34% (6.0 km/h) Hip: 4% Wrist: 17% (self-selected comfort speed)
Jones et al. 2018 [56]	GT3X +	Manually counted	Pearson correlation coefficient	0.997 (8 km/h) 0.998 (10 km/h) 0.990 (12 km/h) 0.905 (14 km/h) 0.762 (16 km/h)
Imboden et al. 2018 [55]	GT3X +	Manually counted	Percentage of bias, Bland–Altman analyses, correlation analysis	Bias: − 32% Correlation coefficient: 0.85 (NA)
Hergenroeder et al. 2018 [50]	GT3X +	Manually counted	Mean measurement bias scores, percentage accuracy	14.1% (< 0.6 m/s) 35.6% (0.60–0.79 m/s) 52.7% (0.80–1.0 m/s) 85.1% (> 1.0 m/s)
Kendall et al. 2019 [11]	wGT3X − BT	Manually counted	Intra-class correlation coefficient	0.919 CI: 0.991–0.996 [incremental test (NA)]
Höchsmann et al. 2020 [58]	GT3X +	StepWatch monitor	Mean absolute percentage errors (MAPE), Intraclass Correlation Coefficient (ICC), 95% confidence intervals (CI), Bland–Altman analyses	Hip: 29% error Wrist: 14% [self-selected comfort speed (NA)]
Bezuidenhout et al. 2021 [61]	GT3X +	StepWatch monitor	Mean percentage agreement, ICC, Bland–Altman analyses	Hip: 0.0 (N); 0.5 (LFE) Ankle: 0.29 (N); 0.97 (LFE) (0.2–0.6 m/s) Hip: 0.0 (N); 0.86 (LFE) Ankle: 0.79 (N); 0.83 (LFE) (0.61–1.0 m/s) Hip: 0.58 (N); 0.87 (LFE) Ankle: 0.85 (N); 0.86 (LFE) (1.1–1.4 m/s) Hip: 0.42 (N); 0.57 (LFE) Ankle: 0.70 (N); 0.70 (LFE) (> 1.4 m/s)
Taoum et al. 2021 [60]	wGT3X +	GPS (for distance), Manually counted (for step count)	Bias of estimation, typical error of estimate (TEE), coefficient of variation, mean percent error (MPE), mean absolute percent error (MAPE)	Hip Step count: 97.8%; CI: 95–99 (N) 99.6; CI: 98–100 (LFE) Distance (MAPE (SD): VM counts: 12.5 (8.5)^a^ and 10 (7.4)^b^ (N); 11.9 (7.4)^a^ and 10.6 (8.2)^b^ (LFE) VM raw data: 12.5 (7.9)^a^ and 8.4 (6.3)^b^ Steps: 17.4 (9.7)^a^ and 18.3 (10.7)^b^ (N); 18.8 (10.3)^a^ and 18.3 (11.3)^b^ (LFE)
Karaca et al. 2021 [59]	wGT3X − BT	Video recordings	Dependent t-test, Pearson correlation coefficient, Bland–Altman analyses, mean absolute percentage error (MAPE)	Hip: 80.0% Wrist: 41.7% (right); 32.3% (left) Ankle: 12.4% (2 km/h) Hip: 8.3% Wrist: 16.3% (right); 26.5% (left) Ankle: 1.0% (4 km/h) Hip: 1.2% Wrist: 25.1% (right); 38.3% (left) Ankle: 4.9% (6 km/h) Hip: 0.8% Wrist: 49.2% (right); 48.6% (left) Ankle: 47.7% (8 km/h) Hip: 2.9% Wrist: 50.9% (right); 51.5% (left) Ankle: 50.6% (10 km/h)

*N* normal filter, *LFE* low frequency extension, *CI* confidence interval, *NA* not applicable, *GPS* global positioning system, *VM* vector magnitude, *NR* non reported

^a^outcome measures yielded by linear mixed models (LMMs); ^b^outcome measures yielded by “speed × time” equation

**Table 6:** Calculation of Cohen’s d

Authors	ActiGraph devices	Outcomes (speed)	Cohen’s d (speed)
Esliger et al. 2007 [52]	7164	5.3% percent difference (50 m/min) 0.008% percent difference (83 m/min) 0.006% percent difference (133 m/min)	1.66 (50 m/min) 1.0 (83 m/min) 0.5 (133 m/min)
Motl et al. 2011 [22]	7164	97.2%; error: 2.8% (54 m/min) 100%; error: 0% (80 m/min) 100.4%; error: + 0.4% (107 m/min)	0.37 (54 m/min) 0 (80 m/min) 0.07 (107 m/min)
Feito et al. 2015 [25]	GT3X + et GT1M	GT1M: − 61% (N); − 7% (LFE) GT3X: − 58% (N); − 4% (LFE) (40 m/min) GT1M: − 31% (N); − 1% (LFE) GT3X: − 31% (N); 1% (LFE) (54 m/min) GT1M: − 7% (N); − 2% (LFE) GT3X: − 6% (N); 2% (LFE) (67 m/min) GT1M: − 6% (N); − 2% (LFE) GT3X: − 1% (N); 3% (LFE) (80 m/min) GT1M: − 9% (N); − 2% (LFE) GT3X: − 2% (N); 3% (LFE) (94 m/min)	GT1M: 2.7 (N); 0.3 (LFE) GT3X: 2.5 (N); 0.2 (LFE) (40 m/min) GT1M: 1.24 (N); 0.04 (LFE) GT3X: 1.34 (N); 0.11 (LFE) (54 m/min) GT1M: 0.32 (N); 0.09 (LFE) GT3X: 0.46 (N); 1.0 (LFE) (67 m/min GT1M: 2.27 (N); 0.09 (LFE) GT3X: 0.14 (N); 1.0 (LFE) (80 m/min) GT1M: 0.31 (N); 0.09 (LFE) GT3X: 0.18 (N); 3.0 (LFE) (94 m/min)
Lee et al. 2015 [27]	GT3X +	0.29 CI: − 0.30–0.62 (54 m/min) 0.33 CI: − 0.25–0.63 (67 m/min) 0.61 CI: 0.28 to 0.79 (80 m/min) 0.99 CI: 0.98–0.99 (94 m/min) 0.99 CI: 0.98–0.99 (107 m/min)	3.35 (54 m/min) 0.81 (67 m/min) 0.52 (80 m/min) 0.001 (94 m/min) 0.001 (107 m/min)
Hickey et al. 2016 [54]	GT3X + et 7164	7164: − 13%; CI: − 19% to -6% GT3X (N): − 54% CI: − 65% to − 42% GT3X (LFE): 1% CI: − 9–11% (2.4 km/h) 7164: − 5%; CI: -6% to − 5% GT3X (N): − 2% CI: − 3% to − 2% GT3X (LFE): − 1% CI: − 1% to − 0.4% (4.8 km/h) 7164: − 5%; CI: − 6% to − 4% GT3X (N): − 2% CI − 2.6% to − 1.7% GT3X (LFE): − 1% CI: − 1% to − 0.4% (7.2 km/h) 7164: − 5% CI: − 6% to − 2% GT3X (N): − 3% CI: − 3% to − 2% GT3X (LFE): − 1% CI: − 2% to − 0.4% (9.7 km/h)	7164: 1.11 GT3X 2.4 (N): GT3X 0.07 (LFE): (2.4 km/h) 7164: 1.18 GT3X 0.45 (N): GT3X 0.2 (LFE): (4.8 km/h) 7164: 0.7 GT3X 0.26 (N): GT3X 0.11 (LFE): (7.2 km/h) 7164: 0.34 GT3X 0.19 (N): GT3X 0.05 (LFE): (9.7 km/h)
Webber et al. 2016 [16]	GT3X +	Ankle (LFE) 0.94 CI: 0.87–0.97 Ankle (N) 0.68 CI: − 0.21 to 0.89 Hip (LFE) 0.83 CI: 0.33–0.94 Hip (N) − 0.05 CI: − 0.19–0.15	Ankle (LFE): 0 Ankle (N): 0.65 (0.4 ± 0.2 m/s) Hip (LFE): 0.14 Hip (N): 0.90 (0.4 ± 0.2 m/s)
Chow et al. 2017 [51]	GT3X +	Hip: − 0.1% Wrist: − 28.9% (5 km/h) Hip: 0.9% Wrist: − 36.0% (6.5 km/h) Hip: − 2.4% Wrist: − 48.4% (8 km/h) Hip: − 0.1% Wrist: − 49.9% (10 km/h) Hip: 0.2% Wrist: − 50.0% (12 km/h)	Hip: 0.16 Wrist: 1.92 (5 km/h) Hip: 0.19 Wrist: 2.49 (6.5 km/h) Hip: 0.35 Wrist: 8.64 (8 km/h) Hip: 0.07 Wrist: 62.37 (10 km/h) Hip: 0.4 Wrist: 125 (12 km/h)
Feng et al. 2017 [53]	wGT3X − BT	− 4% CI: -9% to 3% (0.9 m/s) − 2.5% CI: -12% to 0.8% (1.1 m/s) − 0.3% CI: -0.8% to 0.8% (1.3 m/s)	1.02 (0.9 m/s) 0.49 (1.1 m/s) 0.07 (1.3 m/s)
Hochsmann et al. 2018 [26]	wGTX +	Hip: 82% Wrist: 47% (1.6 km/h) Hip: 24% Wrist: 22% (3.2 km/h) Hip: < 3% Wrist: 30% (4.8 km/h) Hip: < 3% Wrist: 34% (6.0 km/h) Hip: 4% Wrist: 17% (self-selected comfort speed)	Hip: 4.67 Wrist: 2.34 (1.6 km/h) Hip: 1.06 Wrist: 1.17 (3.2 km/h) Hip: 0.27 Wrist: 1.85 (4.8 km/h) Hip: 0.19 Wrist: 2.12 (6.0 km/h) Hip: 0.24 Wrist: 1.13 (self-selected comfort speed)
Jones et al. 2018 [56]	GT3X +	0.997 (8 km/h) 0.998 (10 km/h) 0.990 (12 km/h) 0.905 (14 km/h) 0.762 (16 km/h)	0 (8 km/h) 0 (10 km/h) 0.19 (12 km/h) 0.21 (14 km/h) 0.3 (16 km/h)
Imboden et al. 2018 [55]	GT3X +	Bias: -32% Correlation coefficient: 0.85	1.84
Kendall et al. 2019 [11]	WGT3X − BT	0.919 CI: 0.991 to 0.996 (incremental test)	0.01 (incremental test (NA))
Höchsmann et al. 2020 [58]	GT3X +	Hip: 29% error Wrist: 14% (self-selected comfort speed)	Hip: 1.26 Wrist: 0.02 (self-selected comfort speed (NA))

*LFE* low frequency extension, *CI* confidence interval, *N* normal filter, *NA* not applicable

### Criterion validity of ActiGraph types for distance

One study estimated distance using the ActiGraph wGT3X + in comparison with global positioning system (GPS) for a total duration of 30 min in outdoor setting and reported a moderate criterion validity [60]. The ActiGraph wGT3X + was positioned on the hip and wrist. However, only outcomes of the hip-worn ActiGraph were reported based on two methods (linear mixed models and equation estimated speed multiplied by time) for distance estimation. The linear mixed models were used to estimate distance from corresponding parameters measured by each activity monitor for each walking bout (GPS distance, hip- and wrist-worn ActiGraph total vector magnitude (VM) raw data, hip- and wrist-worn ActiGraph total VM counts and total steps. VM raw data and counts refer to the VM computed from the resampled raw acceleration and counts per second for a yielded wearing positioning [60]. The equation estimated speed was based on each walking bout (GPS mean speed, hip- and wrist-worn ActiGraph mean VM raw data, hip- and wrist-worn ActiGraph mean VM counts and step cadence, and ankle-worn StepWatch step cadence). A walking bout was defined as period of time in which steps occurred in subsequent 30-s intervals. For each method, distance estimation was assessed from vector magnitude (defined by $\sqrt{{x}^{2}+{y}^{2}+{z}^{2}}$ where x, y, and z represent the raw acceleration or the counts yielded from each axis) counts, vector magnitude raw data and total steps [60]. Outcomes seem to show use of vector magnitude counts and vector magnitude raw data is more accurate than use of steps for both distance estimation methods (linear mixed models and equation estimated speed multiplied by time) [60].

## Discussion

The main objective of this systematic review was to determine the criterion validity of ActiGraph devices for step counting and distance estimation in healthy adults and older adults. Twenty-one articles were included in this review and results showed that the ActiGraph GT3X + and wGT3X − BT yielded better criterion validity than the ActiGraph 7164, wGTX + and GT1M for step counting. One study examined the criterion validity of ActiGraph wGT3X + for the estimation of distance travelled in adults.

Studies included in this systematic review evaluated the criterion validity of ActiGraph devices for step counting and distance in adults and elderly people. Five different ActiGraph devices were reported and the ActiGraph GT3X +/wGT3X + was predominantly reported in 13 out of 21 studies [1, 16, 25, 27, 50, 51, 54–61]. All ActiGraph devices reported in this systematic review included only the accelerometer and assessed in indoor setting except in two studies (outdoor setting) [58, 60]. Furthermore, assessment time was short (from 2 to 15 min) in most studies with small errors. For example, Esliger et al. [52] reported 5.3% of error on four minutes of walking (i.e. five to seven steps per minute). Thus, results did not reflect daily use of the ActiGraph devices in outdoor setting in healthy people.

### Criterion validity according to the ActiGraph devices

#### For step counting

Overall, the criterion validity of ActiGraph is distinguished by type of internal accelerometer (unidirectional or tridirectional) and different analysis algorithms. Two unidirectional ActiGraph devices (the ActiGraph 7164 and the ActiGraph GT1M) showed a moderate criterion validity. Indeed, the ActiGraph 7164 was valid (≤ 5.3% error) in two studies [22, 52] and according to walking speeds, had a moderate (≤ 13% error) validity in one study [54]. The ActiGraph GT1M exhibited low to high validity (0.37 ≤ r ≤ 0.69) depending on walking speeds in the study by Abel et al. [24] and (− 61% ≤ difference ≤ − 1%) in the study by Feito et al. [25]. These results are not encouraging for the use of these two unidirectional ActiGraph devices for step counting. Regarding the tridirectional devices, the ActiGraph wGTX + was partially valid in the only study that had evaluated it [26], while the validity of ActiGraph GT3X +/wGT3X + was good in the most studies, except in four studies that had reported validities (from low to high) according to walking speeds [16, 27, 57, 61]. Indeed, step count validity was low at low walking speeds (≤ 0.9 m/s) and good to excellent at self-selected comfort walking or running speeds (≤ 4.44 m/s) [16, 50, 57]. The ActiGraph wGT3X − BT yielded high criterion validity at walking speeds (from 0.9 m/s to 1.3 m/s) in three studies that assessed it [11, 25, 60].

### For distance estimation

Only the hip-worn ActiGraph wGT3X + was used in one study [60]. Therefore, a comparison of criterion validity of ActiGraph types is not possible for distance estimation. In this study, two methods were used based on linear mixed models (method 1) and equation estimated speed multiplied by time (method 2) from vector magnitude counts, vector magnitude raw data and steps. Overall, method 2 seems to yield outcomes of distance estimation more accurate than method 1 regardless of data used. However, one study is insufficient to make conclusion.

### Criterion validity depending on filters used

Filters significantly impact on the criterion validity of the ActiGraph devices. Indeed, in individuals with low walking speeds (e.g. frail elderly), the use of filters (e.g. low frequency extension—LFE with cutoff frequency at 4 Hz, 10 Hz) allows extending the bandwidth, and theoretically increases sensitivity to lower intensity movements [25, 62]. Therefore, the LFE allows to increase the sensitivity of accelerometer signal at low intensity movements by decreasing the proprietary amplitude threshold [61]. However, the LFE seems not to be relevant for step detecting at high intensity movements [61]. Validity of the ActiGraph GT3X + was higher using LFE (e.g. ICC = 0.83) in comparison with default data processing (e.g. ICC = 0.05) independently of the positioning (hip, ankle) in slow walkers [16]. However, in individuals with high walking speeds, a LFE can lead to an overestimating of actual steps due to greater amount of movement artifacts being counted as steps, specifically for the wrist-worn ActiGraph [63]. A LFE seems not to improve accuracy the ActiGraph wGT3X + for distance estimation in adults with self-selected walking speed [60].

### Criterion validity depending on sampling frequency

Nine studies did not report signal processing, however signal processing can affect outcomes. For studies which reported signal processing, sampling frequency was not the same, although the sampling frequency was within frequency range provided by the manufacturer. Indeed, nine studies which assessed the ActiGraph GT3X + /wGT3X + reported three sampling frequencies (30 Hz, 60 Hz and 100 Hz) [1, 27, 51, 55–58, 60, 61]. Step count validity with sampling frequency of 100 Hz was low (0.03 ≤ ICC ≤ 0.64) in study of Riel et al. [57] and moderate (23% of error) in study of Webber et al. [1]. Three out of five studies using 30 Hz of sampling frequency had step count validity varying of low to high (0.0 ≤ ICC ≤ 0.99 and − 50% ≤ error ≤ − 0.1%) [27, 51, 61]. In two studies, criterion validity was good (0.76 ≤ r ≤ 0.99 [56] and 97.8% ≤ detection rate ≤ 99.6% [60]) for step count using a sampling frequency of 30 Hz. Two studies used a sampling frequency of 60 Hz and reported a moderate (− 32% ≤ error ≤ 14%) validity of step detection [55, 58]. Results of these nine studies did not indicate which sampling frequency was more appropriate for a better step count.

### Criterion validity depending on dynamic range

Two studies used the ActiGraph 7164 with different dynamic ranges (0.05–3.2 g and 0.5–1.25 g) and reported different accuracies [22, 52]. Indeed, in the study of Esliger et al. [52], acceleration with 0.5–1.25 g of dynamic range seemed to yield a better accuracy in detecting steps. Dynamic ranges of 0.06–1.94 g and ± 6 g were reported only in one study for the ActiGraph GT1M [24] and GT3X + [1], respectively. No studies reported dynamic range of ActiGraph wGT3X +. Therefore, it is difficult to assess impact of dynamic range on criterion validity of ActiGraph GT1M, GT3X + and wGT3X +.

### Criterion validity according to walking speed

Results showed the impact of walking or running speeds on the criterion validity of ActiGraph types for step counting. Indeed, slow walking did not allow valid step counting measurements using the ActiGraph devices. Thus, all the ActiGraph devices were not valid for walking speeds below 54 m min^−1^ (0.9 m/s) [10, 24–27, 50, 54, 57]. There is probably a speed threshold value for each ActiGraph device, below which step counting is no longer valid. The signal measured might not be sufficient to reach the proprietary threshold in step detecting for slow walking. Indeed, slow walking is generally characterized by low acceleration amplitude. These results are in accordance with data reported in the literature [29, 30, 39, 64]. Indeed, studies have reported low criterion validity for step counting using activity monitors integrating an accelerometer at low walking speed [10, 30, 39].

### Criterion validity according to the positioning of ActiGraph devices

The criterion validity of the ActiGraph devices also differs depending on the positioning. Indeed, all 21 studies positioned the ActiGraph devices on the hip. Four studies placed the ActiGraph devices on the hip and the wrist simultaneously [26, 51, 58, 60] two studies on the hip and the ankle [16, 61] and one study on the hip, wrist and ankle [59]. All studies that quantified number of steps with the wrist-worn ActiGraph devices used an average comparison and reported significant differences in regards to gold standard. The hip-worn ActiGraph generally showed a better validity depending on walking/running speeds and ActiGraph devices. These results can be explained by the fact that during walking or running, the upper limbs and mainly the wrist generate acceleration movements that can induce false positive or true negative in results of step detection. The hip produces less random movements, which can reduce steps detection biases. A possible reason for this under- or overestimation of steps could be a lack of specificity of signal processing algorithms to differentiate between actual steps and spurious or false positive step detection caused by the bouncing of the accelerometers on the waist belt [52]. The ankle-worn ActiGraph GT3X + and wGT3X − BT seemed to yield outcomes more valid in step detection at walking speeds from 1.1 m/s to 1.6 m/s in two studies which assessed different walking speeds (0.2 m/s to 2.7 m/s) [59, 61]. The only study that compared three wearing positions of ActiGraph wGT3X − BT (hip, wrist and ankle) reported that for step counting, the hip positioning was the most valid at walking speeds from 1.1 m/s to 2.7 m/s [59]. However, in the same study, the hip-worn ActiGraph wGT3X − BT was the less valid at walking speed of 0.5 m/s [59]. Both studies that used the ActiGraph GT3X + located on the hip and ankle reported the ankle-worn ActiGraph GT3X + yielded less error than the hip-worn ActiGraph GT3X + for step counting at walking speeds from 0.2 m/s to over 1.4 m/s [16, 61]. The number of studies and participants is small to conclude on the impact of ActiGraph positioning on results validity. Nonetheless, criterion validity of ActiGraph seemed to depend on walking speed, positioning and ActiGraph types. Results reported in this systematic review conform with the literature on the influence of positioning of activity monitors on validity of step counting [65].

### Strengths and weaknesses of ActiGraph devices

#### For step counting

This systematic review shows that the ankle-worn ActiGraph GT3X + is valid for step counting at walking speeds from 0.2 m/s to over 1.4 m/s in indoor setting. However, only two studies have assessed the ankle-worn ActiGraph GT3X +. Step counting with hip-worn ActiGraph wGT3X − BT also appeared valid, depending on walking speeds (from 1.1 m/s to 2.7 m/s). Therefore, there is a minimum walking speed (0.9 m/s) below which some ActiGraph devices are no longer valid [22, 52]. The ActiGraph GT3X + and wGT3X − BT seem to be the most valid devices for step counting. However, all included studies were conducted in indoor setting except two studies [58, 60]. Therefore, results did not reflect daily use of the ActiGraph devices. In 19 out of 21 studies, the ActiGraph devices were assessed on short durations with small errors. This can lead to large differences over a 24-h period of use. In addition, sample sizes of the studies were small, thus results cannot be generalized. Results showed that some ActiGraph devices were not valid at low walking speeds (< 0.9 m/s) for step counting.

#### For distance estimation

Furthermore, ActiGraph devices provide raw data that need to be analyzed using custom algorithms. The availability of raw data should facilitate development of algorithms for distance estimation. Only one recent study assessed criterion validity of the ActiGraph wGT3X + located on the hip for distance estimation and reported moderate results (7.4–18.8% of error). However, other studies should be realized to estimate distance using different ActiGraph types, ActiGraph locations and walking/running speeds.

### Limitations

This systematic review focused only on studies conducted in adults and older adults to reduce variability of reported data and thus reduce the risk of bias related to variability in walking patterns. However, other systematic reviews should be conducted to identify the psychometric properties of the ActiGraph in pathological populations (e.g., stroke survivors) which have variable walking patterns. In addition, only one study included in this systematic review focused on criterion validity of the ActiGraph devices for the estimation of distance. This can be due to the activity monitor types and the healthy population defined in our inclusion criteria. However, it is important to note a lack of studies on the recent ActiGraph GT9X +, which could also allow a good validity because data analysis is based on various sensors. It should be mentioned that results of the studies included in this systematic review are mostly performed in indoor setting exempt for two studies. However, according to the manufacturer, the main purpose of ActiGraph devices is to collect information in daily life in individuals (e.g., to evaluate their life quality or physical activity practice). Several studies did not report the signal processing, sensitivity, dynamic range and analysis algorithm. Indeed, the signal processing needs to be reported in studies to facilitate comparison of devices. A design standardized validation protocol should be established to normalize validation method and enable comparison between devices. The design standardized validation protocol should indicate different walking or running speeds, durations and settings of assessment, signal processing description, device location, etc.

## Conclusion

The main objective of this systematic review was to determine the criterion validity of ActiGraph devices for step counting and distance estimation in healthy adults and older adults. This review revealed a lack of studies (only one study) on the estimation of distance travelled in healthy people. The hip-worn ActiGraph wGT3X + yields a moderate criterion validity for distance estimation. Regarding the criterion validity for the step count, this systematic review revealed that the ActiGraph GT3X + /wGT3X + and wGT3X − BT provide outcomes that are closer to reference measures than other previous ActiGraph devices. Results showed that the ActiGraph GT3X + /wGT3X + and wGT3X − BT have good criterion validity for step counting (under certain conditions of walking speed, positioning and filters used).

## Supplementary Information

**Additional file 1. **Search strategies for all databases. Ti: title; ab: abstract; kw: keyword; MH: exact subject heading; WN KY: Subject/Title/Abstract.

## Data Availability

Not applicable.

## Abbreviations

CI: Confidence interval
DR: Dynamic range
GPS: Global positioning system
Hz: Hertz
ICC: Interclass correlation coefficient
LMM: Linear mixed models
LFE: Low frequency extension
MAPE: Mean absolute percentages error
MEMS: Microelectromechanical system
N: Normal filter
SF: Sampling frequency
SD: Standard deviation
VM: Vector magnitude

## References

[1] Webber SC, Magill SM, Schafer JL, Wilson KC (2014). GT3X+ accelerometer, Yamax pedometer and SC-StepMX pedometer step count accuracy in community-dwelling older adults. J Aging Phys Act.

[2] Hirvensalo M, Rantanen T, Heikkinen E (2000). Mobility difficulties and physical activity as predictors of mortality and loss of independence in the community-living older population. J Am Geriatr Soc.

[3] Marchiori C, Bensmail D (2015). Manual wheelchair satisfaction among long-term users and caregivers: a French study. J Rehabil Res Dev.

[4] Pettersson I, Hagberg L, Fredriksson C, Hermansson LN (2016). The effect of powered scooters on activity, participation and quality of life in elderly users. Disabil Rehabil Assist Technol.

[5] Watson KB, Frederick GM, Harris CD, Carlson SA, Fulton JE (2015). US adults’ participation in specific activities: behavioral risk factor surveillance system—2011. J Phys Act Health.

[6] Garber CE, Blissmer B, Deschenes MR, Franklin BA, Lamonte MJ, Lee I-M, et al. Quantity and quality of exercise for developing and maintaining cardiorespiratory, musculoskeletal, and neuromotor fitness in apparently healthy adults: guidance for prescribing exercise. 2011. 10.1249/MSS.0b013e318213fefb 21694556

[7] Webber SC, Porter MM, Menec VH (2010). Mobility in older adults: a comprehensive framework. Gerontologist.

[8] Feeny DH, Eckstrom E, Whitlock EP, Perdue LA. A primer for systematic reviewers on the measurement of functional status and health-related quality of life in older adults. 2013. 24199257

[9] Organization WH. Towards a Common Language for Functioning, Disability and Health: ICF. The International Classification of Functioning, Disability and Health: World Health Organization; 2002.

[10] Storti KL, Pettee KK, Brach JS, Talkowski JB, Richardson CR, Kriska AM (2008). Gait speed and step-count monitor accuracy in community-dwelling older adults. Med Sci Sports Exerc.

[11] Kendall B, Bellovary B, Gothe NP (2019). Validity of wearable activity monitors for tracking steps and estimating energy expenditure during a graded maximal treadmill test. J Sports Sci.

[12] Farina N, Lowry RG (2018). The validity of consumer-level activity monitors in healthy older adults in free-living conditions. J Aging Phys Act.

[13] Degroote L, De Bourdeaudhuij I, Verloigne M, Poppe L, Crombez G (2018). The accuracy of smart devices for measuring physical activity in daily life: validation study. JMIR Mhealth Uhealth.

[14] Albaum E, Quinn E, Sedaghatkish S, Singh P, Watkins A, Musselman K (2019). Accuracy of the Actigraph wGT3X − BT for step counting during inpatient spinal cord rehabilitation. Spinal cord.

[15] Anderson JL, Yoward LS, Green AJ (2019). A study investigating the validity of an accelerometer in quantification of step count in adult hospital inpatients recovering from critical illness. Clin Rehabil.

[16] Webber SC, John PDS (2016). Comparison of ActiGraph GT3X+ and StepWatch step count accuracy in geriatric rehabilitation patients. J Aging Phys Act.

[17] Campos C, DePaul VG, Knorr S, Wong JS, Mansfield A, Patterson KK (2018). Validity of the ActiGraph activity monitor for individuals who walk slowly post-stroke. Top Stroke Rehabil.

[18] Polese JC, Faria GS, Ribeiro-Samora GA, Lima LP, de Morais Faria CDC, Scianni AA (2019). Google fit smartphone application or Gt3X Actigraph: which is better for detecting the stepping activity of individuals with stroke? A validity study. J Bodywork Movement Therapies..

[19] Compagnat M, Batcho CS, David R, Vuillerme N, Salle JY, Daviet JC (2019). Validity of the walked distance estimated by wearable devices in stroke individuals. Sensors.

[20] McClain JJ, Sisson SB, Washington TL, Craig CL, Tudor-Locke C (2007). Comparison of Kenz Lifecorder EX and Actigraph accelerometers in 10-yr-old children. Med Sci Sports Exerc.

[21] Xi Y, Russell J, Zhang Q, Wang YY, Zhang J, Zhao WH (2019). Validity and reliability of the wristband activity monitor in free-living children aged 10–17 years. Biomed Environ Sci.

[22] Motl RW, Snook EM, Agiovlasitis S (2011). Does an accelerometer accurately measure steps taken under controlled conditions in adults with mild multiple sclerosis?. Disabil Health J.

[23] Sandroff BM, Motl RW, Pilutti LA, Learmonth YC, Ensari I, Dlugonski D (2014). Accuracy of StepWatch™ and ActiGraph accelerometers for measuring steps taken among persons with multiple sclerosis. PLoS ONE.

[24] Abel MG, Hannon JC, Sell K, Lillie T, Conlin G, Anderson D (2008). Validation of the Kenz Lifecorder EX and ActiGraph GT1M accelerometers for walking and running in adults. Appl Physiol Nutr Metab.

[25] Feito Y, Garner HR, Bassett DR. Evaluation of ActiGraph’s low-frequency filter in laboratory and free-living environments: DigitalCommons@ Kennesaw State University; 2015. 10.1249/MSS.0000000000000395 24870583

[26] Höchsmann C, Knaier R, Eymann J, Hintermann J, Infanger D, Schmidt-Trucksäss A (2018). Validity of activity trackers, smartphones, and phone applications to measure steps in various walking conditions. Scand J Med Sci Sports.

[27] Lee JA, Williams SM, Brown DD, Laurson KR (2015). Concurrent validation of the Actigraph gt3x+, Polar Active accelerometer, Omron HJ-720 and Yamax Digiwalker SW-701 pedometer step counts in lab-based and free-living settings. J Sports Sci.

[28] Bassett DR, Ainsworth BE, Leggett SR, Mathien CA, Main JA, Hunter DC (1996). Accuracy of five electronic pedometers for measuring distance walked. Med Sci Sports Exerc.

[29] Hendelman D, Miller K, Baggett C, Debold E, Freedson P (2000). Validity of accelerometry for the assessment of moderate intensity physical activity in the field. Med Sci Sports Exerc.

[30] Tsang K, Dicianno BE (2016). Validity of activity monitors in wheelchair users: a systematic review. J Rehabil Res Dev.

[31] Hänggi JM, Phillips LR, Rowlands AV (2013). Validation of the GT3X ActiGraph in children and comparison with the GT1M ActiGraph. J Sci Med Sport.

[32] Cain KL, Conway TL, Adams MA, Husak LE, Sallis JF (2013). Comparison of older and newer generations of ActiGraph accelerometers with the normal filter and the low frequency extension. Int J Behav Nutr Phys Act.

[33] Vanhelst J, Mikulovic J, Bui-Xuan G, Dieu O, Blondeau T, Fardy P (2012). Comparison of two ActiGraph accelerometer generations in the assessment of physical activity in free living conditions. BMC Res Notes.

[34] Straiton N, Alharbi M, Bauman A, Neubeck L, Gullick J, Bhindi R (2018). The validity and reliability of consumer-grade activity trackers in older, community-dwelling adults: a systematic review. Maturitas.

[35] O'Brien MW, Wojcik WR, Fowles JR (2018). Medical-grade physical activity monitoring for measuring step count and moderate-to-vigorous physical activity: validity and reliability study. JMIR Mhealth Uhealth.

[36] O’Neill B, McDonough S, Wilson J, Bradbury I, Hayes K, Kirk A (2017). Comparing accelerometer, pedometer and a questionnaire for measuring physical activity in bronchiectasis: a validity and feasibility study. Respir Res.

[37] Hart TL, Brusseau T, Kulinna PH, McClain JJ, Tudor-Locke C (2011). Evaluation of low-cost, objective instruments for assessing physical activity in 10–11-year-old children. Res Q Exerc Sport.

[38] Ferguson T, Rowlands AV, Olds T, Maher C (2015). The validity of consumer-level, activity monitors in healthy adults worn in free-living conditions: a cross-sectional study. Int J Behav Nutr Phys Act.

[39] Fuller D, Colwell E, Low J, Orychock K, Tobin MA, Simango B (2020). Reliability and validity of commercially available wearable devices for measuring steps, energy expenditure, and heart rate: systematic review. JMIR Mhealth Uhealth.

[40] Moher D, Liberati A, Tetzlaff J, Altman DG, Group PRISMA (2009). Preferred reporting items for systematic reviews and meta-analyses: the PRISMA statement. Ann Internal Med..

[41] John D, Freedson P (2012). ActiGraph and Actical physical activity monitors: a peek under the hood. Med Sci Sports Exerc.

[42] Terwee CB, Mokkink LB, Knol DL, Ostelo RW, Bouter LM, de Vet HC (2012). Rating the methodological quality in systematic reviews of studies on measurement properties: a scoring system for the COSMIN checklist. Qual Life Res.

[43] MacDermid J (2008). Critical appraisal of study design for psychometric articles evaluation form and interpretation guide. Evidence based rehabilitation: a guide to practice.

[44] Balshem H, Helfand M, Schünemann HJ, Oxman AD, Kunz R, Brozek J (2011). GRADE guidelines: 3. Rating the quality of evidence. J Clin Epidemiol.

[45] Gwet KL. Handbook of inter-rater reliability: the definitive guide to measuring the extent of agreement among raters: Advanced Analytics, LLC; 2014.

[46] Koo TK, Li MY (2016). A guideline of selecting and reporting intraclass correlation coefficients for reliability research. J Chiropr Med.

[47] Cohen A, Schagerlof H, Nilsson C, Melander C, Tjerneld F, Gorton L (2004). Liquid chromatography-mass spectrometry analysis of enzyme-hydrolysed carboxymethylcellulose for investigation of enzyme selectivity and substituent pattern. J Chromatogr A.

[48] Cicchetti DV (1994). Guidelines, criteria, and rules of thumb for evaluating normed and standardized assessment instruments in psychology. Psychol Assess.

[49] Hopkins WG, Marshall SW, Batterham AM, Hanin J. Progressive statistics for studies in sports medicine and exercise science. LWW; 2009. 10.1249/MSS.0b013e31818cb278 19092709

[50] Hergenroeder AL, Barone Gibbs B, Kotlarczyk MP, Kowalsky RJ, Perera S, Brach JS (2018). Accuracy of objective physical activity monitors in measuring steps in older adults. Gerontol Geriatr Med.

[51] Chow JJ, Thom JM, Wewege MA, Ward RE, Parmenter BJ (2017). Accuracy of step count measured by physical activity monitors: the effect of gait speed and anatomical placement site. Gait Posture.

[52] Esliger DW, Probert A, Gorber SC, Bryan S, Laviolette M, Tremblay MS (2007). Validity of the actical accelerometer step-count function. Med Sci Sports Exerc.

[53] Feng Y, Wong CK, Janeja V, Kuber R, Mentis HM (2017). Comparison of tri-axial accelerometers step-count accuracy in slow walking conditions. Gait Posture.

[54] Hickey A, John D, Sasaki JE, Mavilia M, Freedson P (2016). Validity of activity monitor step detection is related to movement patterns. J Phys Act Health.

[55] Imboden MT, Nelson MB, Kaminsky LA, Montoye AH (2018). Comparison of four Fitbit and Jawbone activity monitors with a research-grade ActiGraph accelerometer for estimating physical activity and energy expenditure. Br J Sports Med.

[56] Jones D, Crossley K, Dascombe B, Hart HF, Kemp J (2018). Validity and reliability of the Fitbit Flex™ and ActiGraph Gt3x+ at jogging and running speeds. Int J Sports Phys Ther.

[57] Riel H, Rathleff CR, Kalstrup PM, Madsen NK, Pedersen ES, Pape-Haugaard LB (2016). Comparison between Mother, ActiGraph wGT3X − BT, and a hand tally for measuring steps at various walking speeds under controlled conditions. PeerJ.

[58] Hochsmann C, Knaier R, Infanger D, Schmidt-Trucksass A (2020). Validity of smartphones and activity trackers to measure steps in a free-living setting over three consecutive days. Physiol Meas.

[59] Karaca A, Demirci N, Yılmaz V, Hazır Aytar S, Can S, Ünver E. Validation of the ActiGraph wGT3X − BT accelerometer for step counts at five different body locations in laboratory settings. Meas Phys Educ Exerc Sci. 2021:1–10.

[60] Taoum A, Chaudru S, de Müllenheim P-Y, Congnard F, Emily M, Noury-Desvaux B, et al. Comparison of activity monitors accuracy in assessing intermittent outdoor walking. Med Sci Sports Exerc. 2021. 10.1249/MSS.0000000000002587 33731660

[61] Bezuidenhout L, Thurston C, Hagströmer M, Moulaee CD (2021). Validity of hip and ankle worn ActiGraph accelerometers for measuring steps as a function of gait speed during steady state walking and continuous turning. Sensors.

[62] Rothney MP, Apker GA, Song Y, Chen KY (2008). Comparing the performance of three generations of ActiGraph accelerometers. J Appl Physiol.

[63] Toth LP, Park S, Springer CM, Feyerabend MD, Steeves JA, Bassett DR (2018). Video-recorded validation of wearable step counters under free-living conditions. Med Sci Sports Exerc.

[64] Cyarto EV, Myers A, Tudor-Locke C (2004). Pedometer accuracy in nursing home and community-dwelling older adults. Med Sci Sports Exerc.

[65] Brognara L, Palumbo P, Grimm B, Palmerini L (2019). Assessing gait in Parkinson’s disease using wearable motion sensors: a systematic review. Diseases.

