# Supplementary material for: Criterion validity of ActiGraph monitoring devices for step counting and distance measurement in adults and older adults: a systematic review
Source: J Neuroeng Rehabil. 2022 Oct 17;19:112. doi: 10.1186/s12984-022-01085-5 (PMC9575229; doi:10.1186/s12984-022-01085-5)
Supplement: Supplementary file 1 — Additional file 1. Search strategies for all databases. Ti: title; ab: abstract; kw: keyword; MH: exact subject heading; WN KY: Subject/Title/Abstract. [file 12984_2022_1085_MOESM1_ESM.docx]

**Supplementary Material**

Table S1: Search strategies for all databases

| Medline(Ovid) | |
| --- | --- |
| 1 | ActiGraph or Actigraph or actigraph or ACTIGRAPGH |
| 2 | Step* count* or detect*of step* or stride count* or detect* of stride* or number of step* or number of stride* or quantificat* of step* or quantificat* of stride* or distance |
| 3 | valid* or accura* or precision or accuracy or accurate or validity or validation |
| 4 | #1 and #2 and #3 |
| 5 | ((ActiGraph or Actigraph or actigraph or ACTIGRAPGH) and (Step* count* or detect*of step* or stride count* or detect* of stride* or number of step* or number of stride* or quantificat* of step* or quantificat* of stride* or distance) and (valid* or accura* or precision or accuracy or accurate or validity or validation)). ti,ab,kw. |

| Embase | |
| --- | --- |
| 1 | ('ActiGraph' or 'Actigraph' or 'actigraph' or 'ACTIGRAPGH'):ti,ab,kw |
| 2 | ('Step* count*' or 'detect*of step*' or 'stride count*' or 'detect* of stride*' or 'number of step*' or 'number of stride*' or 'quantificat* of step*' or 'quantificat* of stride*' or 'distance'): ti,ab,kw |
| 3 | ('valid*' or 'accura*' or 'precision' or 'accuracy' or 'accurate' or 'validity' or 'validation'): ti,ab,kw |
| 4 | #1 and #2 and #3 |
| 5 | (('ActiGraph' or 'Actigraph' or 'actigraph' or 'ACTIGRAPGH') and ('Step* count*' or 'detect*of step*' or 'stride count*' or 'detect* of stride*' or 'number of step*' or 'number of stride*' or 'quantificat* of step*' or 'quantificat* of stride*' or 'distance') and ('valid*' or 'accura*' or 'precision' or 'accuracy' or 'accurate' or 'validity' or 'validation')):ti,ab,kw |

| CINAHL (EBSCO) | |
| --- | --- |
| 1 | AB (ActiGraph or Actigraph or actigraph or ACTIGRAPGH) OR TI (ActiGraph or Actigraph or actigraph or ACTIGRAPGH) |
| 2 | MH (ActiGraph) |
| 3 | #1 or #2 |
| 4 | AB (Step* count* or detect*of step* or stride count* or detect* of stride* or number of step* or number of stride* or quantificat* of step* or quantificat* of stride* or distance) OR TI (Step* count* or detect*of step* or stride count* or detect* of stride* or number of step* or number of stride* or quantificat* of step* or quantificat* of stride* or distance) |
| 5 | MH (step count) or MH (distance) |
| 6 | #4 or #5 |
| 7 | AB (valid* or accura* or precision or accuracy or accurate or validity or validation) OR TI (valid* or accura* or precision or accuracy or accurate or validity or validation) |
| 8 | MH (validity) |
| 9 | #7 or #8 |
| 10 | #3 and #6 and #9 |

| IEEExplore | |
| --- | --- |
| 1 | Full Text only (ActiGraph) |
| 2 | Full Text only (step* count* or detect*of step or number of stride* or distance) |
| 3 | ("Full Text Only": ActiGraph) AND ("Full Text Only":step* count* or detect*of step or number of stride* or distance) |

| Engineering Village | |
| --- | --- |
| 1 | Subject/Title/Abstract (ActiGraph or Actigraph or actigraph or ACTIGRAPGH) |
| 2 | Subject/Title/Abstract (Step* count* or detect*of step* or stride count* or detect* of stride* or number of step* or number of stride* or quantificat* of step* or quantificat* of stride* or distance) |
| 3 | Subject/Title/Abstract (valid* or accura* or precision or accuracy or accurate or validity or validation) |
| 4 | ((((ActiGraph or Actigraph or actigraph or ACTIGRAPGH) WN KY) AND ((Step* count* or detect*of step* or stride count* or detect* of stride* or number of step* or number of stride* or quantificat* of step* or quantificat* of stride* or distance) WN KY)) AND ((valid* or accura* or precision or accuracy or accurate or validity or validation) WN KY)) |

| Web of Science | |
| --- | --- |
| 1 | ("ActiGraph" or "Actigraph" or "actigraph" or "ACTIGRAPGH")(Abstract) |
| 2 | ("ActiGraph" or "Actigraph" or "actigraph" or "ACTIGRAPGH")(Title) |
| 3 | #1 or #2 |
| 4 | ("Step* count*" or "detect*of step*" or "stride count*" or "detect* of stride*" or "number of step*" or "number of stride*" or "quantificat* of step*" or "quantificat* of stride*" or "distance") (Abstract) |
| 5 | ("Step* count*" or "detect*of step*" or "stride count*" or "detect* of stride*" or "number of step*" or "number of stride*" or "quantificat* of step*" or "quantificat* of stride*" or "distance") (Title) |
| 6 | #4 or #5 |
| 7 | ("valid*" or "accura*" or "precision" or "accuracy" or "accurate" or "validity" or "validation") (Abstract) |
| 8 | ("valid*" or "accura*" or "precision" or "accuracy" or "accurate" or "validity" or "validation") (Title) |
| 9 | #7 or #8 |
| 10 | #3 and #6 and #9 |
